# Commensal microbiota modulate gene expression in the skin

**DOI:** 10.1186/s40168-018-0404-9

**Published:** 2018-01-30

**Authors:** Jacquelyn S. Meisel, Georgia Sfyroera, Casey Bartow-McKenney, Ciara Gimblet, Julia Bugayev, Joseph Horwinski, Brian Kim, Jonathan R. Brestoff, Amanda S. Tyldsley, Qi Zheng, Brendan P. Hodkinson, David Artis, Elizabeth A. Grice

**Affiliations:** 1 Department of Dermatology, University of Pennsylvania Perelman School of Medicine, 421 Curie Blvd, 1015 BRB II/III, Philadelphia, PA 19104 USA; 2 Department of Dermatology, Washington University School of Medicine, St. Louis, MO 63110 USA; 3 Department of Pathology and Immunology, Washington University School of Medicine, St. Louis, MO 63110 USA; 4 Jill Roberts Institute for Research in Inflammatory Bowel Disease, Joan and Sanford I. Weill Department of Medicine, Weill Cornell Medicine, Cornell University, New York, NY 10021 USA

**Keywords:** RNA sequencing, Cutaneous transcriptome, Germ-free mice, Host-microbe interactions, Skin microbiome

## Abstract

**Background:**

The skin harbors complex communities of resident microorganisms, yet little is known of their physiological roles and the molecular mechanisms that mediate cutaneous host-microbe interactions. Here, we profiled skin transcriptomes of mice reared in the presence and absence of microbiota to elucidate the range of pathways and functions modulated in the skin by the microbiota.

**Results:**

A total of 2820 genes were differentially regulated in response to microbial colonization and were enriched in gene ontology (GO) terms related to the host-immune response and epidermal differentiation. Innate immune response genes and genes involved in cytokine activity were generally upregulated in response to microbiota and included genes encoding toll-like receptors, antimicrobial peptides, the complement cascade, and genes involved in IL-1 family cytokine signaling and homing of T cells. Our results also reveal a role for the microbiota in modulating epidermal differentiation and development, with differential expression of genes in the epidermal differentiation complex (EDC). Genes with correlated co-expression patterns were enriched in binding sites for the transcription factors Klf4, AP-1, and SP-1, all implicated as regulators of epidermal differentiation. Finally, we identified transcriptional signatures of microbial regulation common to both the skin and the gastrointestinal tract.

**Conclusions:**

With this foundational approach, we establish a critical resource for understanding the genome-wide implications of microbially mediated gene expression in the skin and emphasize prospective ways in which the microbiome contributes to skin health and disease.

**Electronic supplementary material:**

The online version of this article (10.1186/s40168-018-0404-9) contains supplementary material, which is available to authorized users.

## Background

As a barrier to the external environment, the skin harbors microbial communities that are both topographically diverse and temporally complex [1–4]. These microbes are postulated to have important functions in skin health [2], including colonization resistance to block invasion of pathogenic bacteria and regulation of the cutaneous inflammatory and immune response [5–7]. The skin must sense, interpret, and respond to microbial signals from the environment, orchestrating responses appropriate for the stimuli while maintaining barrier function and protecting itself from pathogenic infection.

Abnormal host-microbe interactions are associated with cutaneous disorders like atopic dermatitis, acne, and psoriasis [8–14], but the exact mechanisms underlying the microbial contributions to disease development and progression are currently unknown. Identifying the complete range of host functions and pathways evoked by the skin microbiota will improve our understanding of disease pathogenesis and reveal new preventative and therapeutic targets.

The full extent of cutaneous functions regulated by the skin microbiota remains unknown and previous work has focused heavily on characterizing the response of specific pathways to microbial colonization. Recent work in mouse models demonstrates that the commensal microbiota, along with hair follicle morphogenesis, is responsible for recruitment of regulatory T cells during neonatal life [15]. Regulatory T cells additionally establish and mediate immune tolerance to skin commensal bacteria during a defined window of development [16]. Skin commensal bacteria also promote interleukin 1 (IL-1) signaling and effector T cell functions, suggesting a role for the microbiota in driving and/or mediating inflammatory skin disorders [6]. Other work has highlighted the contributions of specific types of bacteria in inducing T cell responses via interactions with skin-resident dendritic cell subsets [17]. Complement, an ancient and evolutionarily conserved arm of the innate immune system, may also be regulated in the skin by colonization with the commensal microbiota [18]. While these studies and others have established roles for the microbiota in shaping cutaneous immunity, the broad spectrum of host functions that are elicited and/or mediated by the microbiota, as well as their underlying molecular mechanisms, remains uncharacterized.

Here, we aimed to identify the molecular signals that mediate the cutaneous host response to the resident skin microbiota on a genome-wide scale, thereby elucidating the full range of cutaneous responses evoked by the microbiome. We used sterile, germ-free mice that have never been exposed to microbiota and compared their cutaneous transcriptome to that of mice conventionally raised in the presence of microbiota. We reasoned that, similar to previous work performed in the gastrointestinal tract [19–25], this experimental design would allow us to identify genes and pathways in the skin under transcriptional modulation by the microbiome.

Differentially expressed genes were enriched for those related to immunity and epidermal differentiation and development. Further analysis revealed an enrichment of microbially regulated genes in the epidermal differentiation complex, a syntenic cluster of genes regulated in a tissue-specific manner with critical roles in epidermal barrier formation [26]. Analysis of coordinately regulated genes suggests that genes under the transcriptional control of Klf-4, AP-1, and SP-1 are microbially regulated. Finally, we identify genes that are similarly regulated by the microbiota in both the skin and gastrointestinal tract, highlighting commonalities in the molecular signals that govern host-microbe interactions at both barrier sites. Collectively, this work provides a critical foundation and resource for understanding cutaneous gene regulation by the microbiota, while establishing the molecular signals governing host responses to microbial colonization.

## Results

### Commensal microbiota modulate the cutaneous transcriptome

**Fig. 1:**
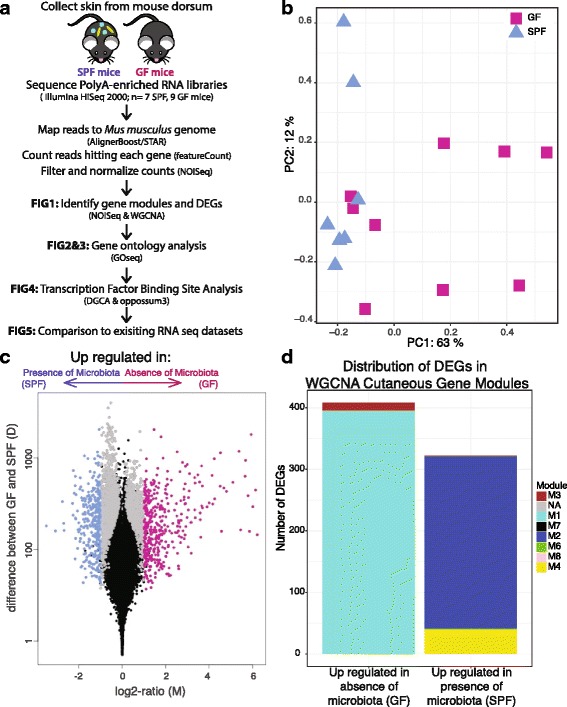
Gene expression profiles differ between SPF and GF skin. **a** Dorsal skin collected from GF and SPF mice was subject to polyA-enriched RNA sequencing to identify transcriptional modulation by skin microbial communities. **b** NMDS plot based on filtered, normalized, batch effect-corrected read counts from each sample, showing that samples cluster together by condition. Blue triangles indicate SPF samples, and magenta squares indicate GF samples. **c** Volcano plot highlighting differentially expressed genes. Each dot represents a gene. Gray dots indicate DEGs. Magenta dots indicate DEGs with at least twofold enrichment in GF mice, while blue dots indicate DEGs with at least twofold enrichment in SPF mice. The *x*-axis is the log fold change in normalized gene expression and the *y*-axis depicts the log_10_ absolute value of the difference in expression between the two conditions. **d** Barplot indicating WGCNA gene modules to which the 730 DEGs with a twofold difference belong

To measure the genome-wide impact of microbial colonization on cutaneous gene transcription, we sequenced and compared the mRNA transcriptome of skin from mice raised in the absence of microbiota to conventionally raised mice (Fig. 1a). Poly-A enriched RNA isolated from murine germ free (GF; *n* = 9) and specific pathogen free, conventionally raised (SPF; *n* = 7) skin was sequenced on the Illumina HiSeq 2000 to obtain over 1.2 billion paired-end reads (median of 60 million reads per sample, see Additional file 1: Table S1 for sample summaries) of good quality (Additional file 2: Figure S1A**)**. Reads were mapped to the mouse reference genome using the STAR aligner [27], in conjunction with AlignerBoost [28]. Of reads that aligned to the mouse reference genome, an average of 88% of reads per sample were assigned to a feature (Additional file 2: Figure S1B), and the majority mapped to protein coding RNA (Additional file 2: Figure S1C) with sufficient coverage (Additional file 2: Figure S1D). Gene counts were filtered and normalized in NOISeq [29, 30], yielding a total of 15,448 features for analysis (Additional file 3: Dataset S1). ARSyNseq [31] was used to control for batch effects associated with different sequencing runs (Additional file 4: Figure S2).

Biological replicates of GF and SPF skin cluster together as demonstrated by non-metric multidimensional scaling (Fig. 1b). A total of 2820 genes were differentially expressed between GF and SPF skin (FDR-corrected *p* value < 0.1, Fig. 1c, Additional file 3: Dataset S1). Of these, 730 genes were differentially expressed by a twofold difference or greater between GF and SPF skin: 408 upregulated and 322 downregulated in the absence of microbiota.

Weighted gene correlation network analysis (WGCNA) [32], an unsupervised method for correlating patterns of gene expression, created a scale-free network with 13 cutaneous gene modules (Fig. 1d, Additional files 5 and 6: Figure S3A-C, Dataset S2). Ninety percent of all genes were assigned to modules, with the majority belonging to modules M1 and M2 (5613 and 5259 genes, respectively). Genes in each module were significantly enriched in different biological processes, including RNA processing and metabolic processes (M1), the immune response (M2), transport (M3), and inflammatory response and keratinocyte differentiation (M4) (Additional file 7: Table S2). While colonization status clustered closely to and was correlated with the M4 module (*ρ* = 0.45, *p* = 0.08), only the M1 (*ρ* = − 0.58, *p* = 0.02) and M2 (*ρ* = 0.6, *p* = 0.01) modules were significantly correlated with the presence of microbiota (Additional file 5: Figure S3D). The majority of differentially expressed genes (DEG) downregulated in SPF skin were assigned to the M1 and M3 modules, while those upregulated in SPF skin were predominantly found in the M2 and M4 modules (Fig. 1d).

### Cutaneous immune response genes are differentially regulated by resident microbiota

**Fig. 2:**
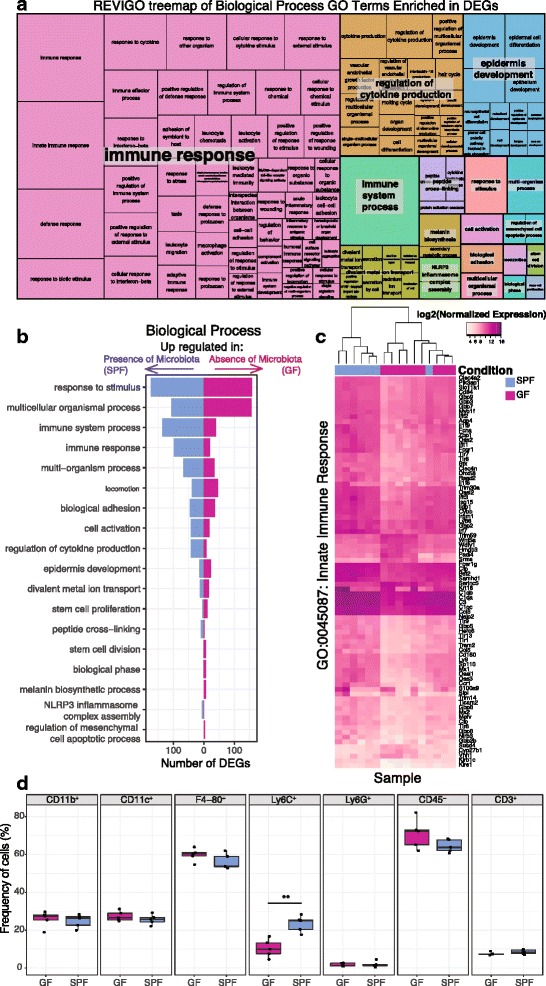
Gene ontology analysis identifies immune response terms enriched in DEGs. **a** REVIGO treemap showing cluster representatives of Biological Process GO terms that are significantly enriched in the DEGs (FDR-corrected *p* value < 0.05). Larger boxes indicate greater significance, as the box sizes are determined by the absolute value of the log_10_ *p* value. **b** Barplot depicting the number of DEGs in each of the high-level significant Biological Process GO terms from part A. **c** Heatmap of the log normalized gene expression of DEGs in the GO term “Innate Immune Response”. **d** Flow cytometry analysis of GF and SPF mice (*n* = 5 each) identified no significant differences between GF and SPF skin in regard to myeloid (CD11b^+^) cells, dendritic (CD11c^+^) cells, macrophages (F4-80^+^), neutrophils (Ly6G^+^), non-hematopoietic (CD45^−^) cells, and T cells (CD3^+^). However, Ly6C^+^ monocytes were significantly increased in frequency in SPF compared to GF skin (*T* test, *p* value < 0.01). All populations (except CD45^−^) were pre-gated on live, CD45^+^ cells

Gene ontology (GO) analysis of the 730 DEGs with an FDR-corrected *p* value > 0.1 and > twofold difference in expression revealed a variety of biological processes modulated by the commensal microbiota (Additional file 8: Dataset S3), including “immune response” (FDR-corrected *p* value 3 × 10^− 24^, Fig. 2a). DEGs contained within GO terms related to the immune response were generally upregulated in SPF skin (Fig. 2b). For instance, of the 428 genes in our dataset that were characterized by the GO term “innate immune response,” 82 are differentially expressed (Fig. 2c). Seventy-two of these “innate immune response” DEGs are upregulated in SPF skin and include genes encoding pattern recognition receptors (*Tlr1*, *Tlr7*, *Tlr8*, *Tlr9 Tlr13*), interferon regulatory factors (*Irf7)*, and the complement cascade (*C3*, *C1qa*, *C1qb*, *C1qc*, *Cfp, Cfb, C3ar1*). DEGs encoding antimicrobial proteins were also upregulated in SPF skin, including *Slpi* and *Ccl6*.

Molecular function GO terms enriched in the DEGs contained terms related to host-microbe interactions and the immune system, such as “cytokine activity” (GO 0005125), “cytokine receptor binding” (GO 0005126), “toll-like receptor binding” (GO 0035325), and “interleukin-1 receptor binding” (GO 0005149) (FDR-corrected *p* values all < 0.05). Analysis of DEGs within the GO term “cytokine activity” revealed differential expression of cytokines/chemokines involved with homing of T cells to the skin, including *Tslp, Cxcl9*, and *Ccl28*, all upregulated in SPF skin. Interleukin-1 family cytokine genes were also upregulated in SPF skin compared to GF, including *IL-1β*, *Il-33*, *Il1f8* (also known as *Il-36β*), *Il1f9* (also known as *Il-36γ*). In particular, IL-36γ has been implicated in plaque psoriasis [33], and Cathepsin S (*Cpss*), recently shown to activate IL-36γ [34], was also upregulated in SPF compared to GF skin. Genes encoding pro-inflammatory cytokines, such as *Il-33*, were upregulated by the microbiota, as were anti-inflammatory cytokines such as *Il-10.*

KEGG (Kyoto Encyclopedia of Genes and Genomes) pathway analysis corroborated enriched GO terms and identified significant enrichment of the pathways “complement and coagulation cascades” (ko04610), “*Staphylococcus aureus* infection” (ko05150), “cytokine-cytokine receptor interaction” (ko04060), and “toll-like receptor signaling pathway” (ko04620) (FDR-corrected *p* value < 0.05).

### Analysis of skin immune cell populations supports gene expression findings

Because the skin is composed of heterogeneous cell populations, and differential infiltration by immune cell subtypes may account for some differences in observed gene expression, we compared GF and SPF skin cellular populations. Toluidine blue staining for mast cells did not reveal significant differences in counts between SPF and GF skin (Additional file 9: Figure S4A), nor did immunofluorescence staining of CD3, a pan T cell marker (Additional file 9: Figure S4B).

Flow cytometry was used to further quantify a variety of different cell populations in the skin of SPF and GF mice. No significant differences were observed between GF and SPF skin in frequency of myeloid (CD11b^+^) cells, dendritic (CD11c^+^) cells, neutrophils (Ly6G^+^), non-hematopoietic (CD45^−^) cells, or T cells (CD3^+^) (Fig. 2d). In SPF skin, however, Ly6C^+^ monocytes were significantly increased in frequency (Fig. 2d). We also saw increased IL-1α production in myeloid, dendritic, macrophage, and non-hematopoietic cell populations in SPF compared to GF skin (Additional file 9: Figure S4C), similar to previous reports of the cutaneous immune microenvironment [6]. Although the frequency of F4/80^+^ macrophages did not differ (Fig. 2d), an increased frequency of IL-1β producing F4/80^+^ macrophages was observed in SPF skin, in line with our RNA sequencing data that identified *IL-1β* as differentially expressed (Additional file 9: Figure S4C, D). Overall, these results support our transcriptome findings of differential gene expression related to cytokine activity and the immune response and confirm previous reports of the cutaneous immune microenvironment.

### Epidermal differentiation is regulated by the commensal microbiota

**Fig. 3:**
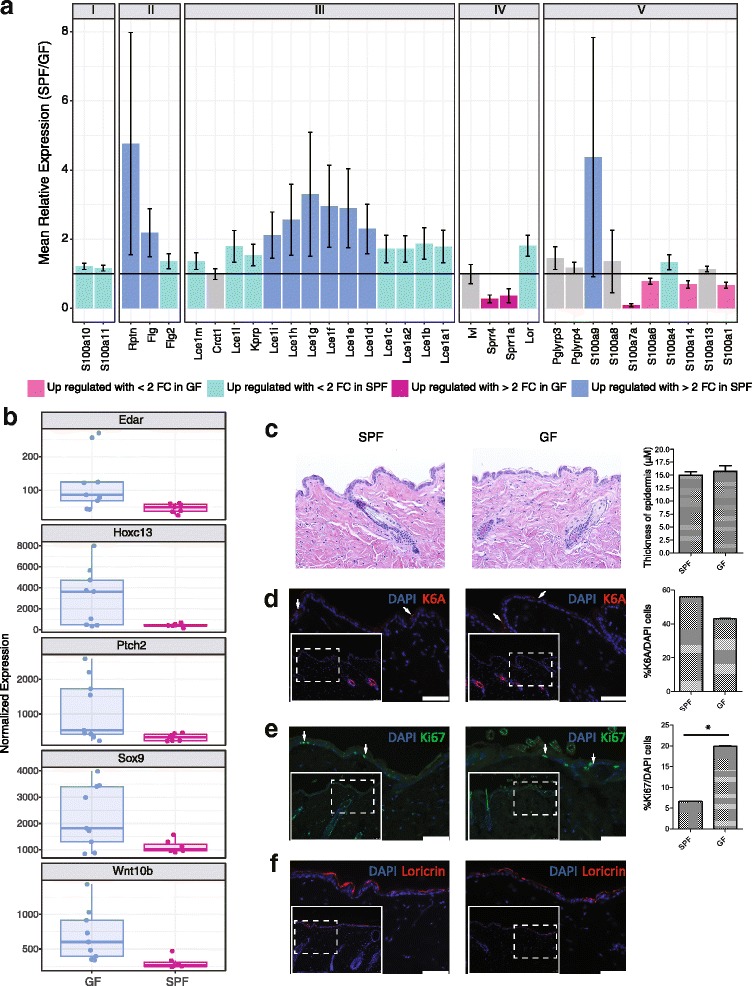
Genes in the epidermal differentiation complex (EDC) are under microbial regulation. **a** The mean relative expression of genes found in the EDC in SPF compared to GF mice. A value of 1 indicates equal expression in the two groups. Colors of the bars indicate DEGs, and error bars represent propagated SE of the ratio SPF/GF. EDC genes are grouped as previously described [35]. **b** Boxplot of normalized gene expression of differentially expressed transcription factors and regulators critical to skin developmental processes. **c***–***f** Histology and immunofluorescence staining of SPF and GF skin sections. Dotted line inset boxes indicate the area that is magnified in the figure to orient the reader. White arrowheads are examples of positive cells. Significance testing was performed on an aggregate of three experiments with 3 GF and 3 SPF mice each. A * indicates a *p* value < 0.05 by *T* test. Scale bars represent 50 μm. **c** Hematoxylin and eosin staining and epidermal thickness measurements. **d** Cytokeratin 6A (K6A) staining. **e** Ki67 staining for proliferating cells. **f** Loricrin staining as a marker of differentiation

The GO term “keratinocyte differentiation” was significantly enriched in DEGs (FDR corrected *p* value 2.2 × 10^− 5^). Of the 101 genes in our dataset that fall under this category, eight were significantly downregulated and 12 were significantly upregulated in response to microbial colonization. Notably, nine of these genes are found in the epidermal differentiation complex (EDC), a cluster of genes found on murine chromosome 3 encoding proteins involved in terminal differentiation and cornification of keratinocytes and implicated in cutaneous diseases such as psoriasis and atopic dermatitis [35]. There are 61 total genes in the murine EDC; 33 were retained in our filtered, normalized dataset, 27 were significantly differentially expressed between GF and SPF mice, and 12 of these DEGs had at least a twofold change in expression (Fig. 3a). This includes late cornified envelope genes (*Lce1d*, *Lce1e*, *Lce1f*, *Lce1g*, *Lce1h*, *Lce1i*, *Lce1j*, *Lce1k*) and small proline rich region genes (*Sprr1a*, *Sprr2a3*, *Sprr4*), which encode cornified envelope precursors with protein cross-linking function, all upregulated in SPF compared to GF skin. Other DEGs localizing to the EDC included those encoding the S100 small calcium binding proteins. These include *S100a7a* (psoriasin) and *S100a9*, both encoding antimicrobial and/or chemotactic proteins which are expressed under a variety of epidermal insults including psoriasis and wound healing [36]. Previous studies have demonstrated that *Escherichia coli*, a Gram-negative bacterium rarely found on human or murine skin, induces expression of S100A7 and S100A15 when heat killed cultures or conditioned media are incubated with keratinocytes in vitro [37, 38]. Additionally, *Flg* and *Rptn* are DEGs encoding the structural proteins filaggrin and repetin, respectively, and were upregulated in SPF mice.

DEGs outside of the EDC but also involved in keratinocyte differentiation included the cell adhesion protein cadherin-3 (*Cdh3*); hornerin (*Hrnr*), a filaggrin-like S100 protein; and keratin 16 (*Krt16*), a structural protein recently shown to regulate innate immunity in response to epidermal barrier stress [39]. Genes upregulated in GF mice included those encoding the transcription factors *Msx2* and *Foxn1*. Notably, *Foxn1* knockout results in the *nude* phenotype, characterized by skin defects including impaired keratinization and hair formation [40], and genetically interacts with *Msx2* upstream of the Notch signaling pathway [41].

The enrichment of DEGs annotated with the “keratinocyte differentiation” GO term prompted us to further examine other gene subsets that are involved in the development and differentiation of the epidermis. This revealed a variety of transcription factors and regulators critical to skin developmental processes including *Ptch2*, *Sox9*, *Edar*, *Wnt10b*, and *Hoxc13*, all of which were upregulated in GF compared to SPF skin (Fig. 3b).

To further investigate these findings and the potential structural consequences to the skin barrier, we assessed gross morphology of SPF and GF skin by performing hematoxylin and eosin staining of histological sections. As shown in Fig. 3c, no differences in the thickness of epidermis or other structural alterations were observed. Immunofluorescence was also used to visualize markers for differentiation, proliferation, and injury. Staining for cytokeratin 6a (K6A) did not differ between SPF and GF mice and was localized to the hair follicle (Fig. 3d), the site of constitutive expression. Since K6A expression in the interfollicular epidermis is a hallmark response to wound healing [42], we conclude that the barrier integrity is similar in SPF and GF mice. Supporting this, the gene encoding K6a (*Krt6a*) was not differentially expressed. Ki-67, a marker of cellular proliferation [43], was significantly increased in GF skin (Fig. 3e), corroborating the finding that the gene encoding Ki-67 (*Mki67*) was also significantly upregulated in GF skin. Loricrin, a major component of the cornified envelope and a marker of keratinocyte terminal differentiation [44], appeared qualitatively to be increased in SPF skin by immunofluorescence (Fig. 3f), suggesting increased terminal differentiation in SPF compared to GF skin. Expression of *Krt1*, another marker of terminal differentiation, was also significantly increased in expression in SPF mice (Additional file 9: Figure S4E); however, there was no significant difference in expression of *Krt14*, expressed primarily by proliferating basal keratinocytes [45]. Together, our transcriptional and histological findings suggest that the balance between epidermal proliferation and differentiation is altered in response to microbial colonization.

### Colonization state shifts gene expression networks for epidermal differentiation and development processes

**Fig. 4:**
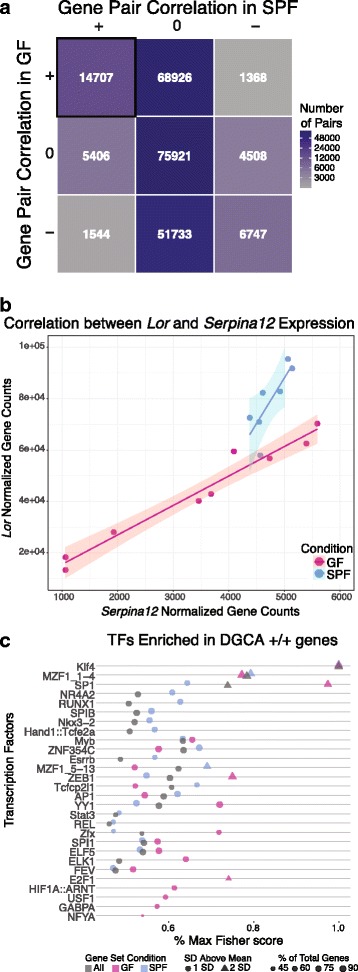
DGCA analysis identified significantly correlated DEGs that share potential transcription factor binding sites. **a** Matrix highlighting the number of significantly correlated gene pairs from the filtered list of DEGs. Each axis represents a condition (GF or SPF), with + indicating a significant positive correlation between the gene pair, − indicating a significant negative correlation, and 0 indicating the lack of a significant correlation. Gene pairs that are positively correlated in both SPF and GF skin are highlighted in the uppermost left corner. **b** The *Loricrin* and *Serpina12* gene pair is positively correlated in both colonization conditions, but a significant loss of correlation is observed in SPF compared to GF skin (*q* < 0.05). The *x*- and *y*-axes indicate the TMM normalized, batch effect-corrected gene counts, and each dot represents a single mouse, colored by their microbial condition. Colored lines and shaded areas represent the linear regression lines and their respective 95% confidence interval for each microbial condition. **c** Analysis with oppossum3 identified enriched transcription factors in positively correlated DGCA gene sets, using Fisher scores to assess significance. The *y*-axis identifies significant transcription factors, while *x*-axis represents the significance metric. Higher values indicate greater significance and the shape indicates whether the metric score was 1 or 2 standard deviations (SD) above the mean. Fisher scores are significant when greater than 1 SD above the mean. Size of each point reflects the percentage of all DGCA +/+ DEGs containing a binding region for each TF and color indicates colonization status of the DGCA +/+ DEGs

To investigate gene-gene regulatory relationships, we identified gene pairs with similar expression patterns in GF and SPF states using differential gene correlation analysis (DGCA) [46]. We focused on a subset of all DEGs with high relative expression, moderate to high dispersion, and significant co-expression patterns across both colonization conditions. Post-filtering, 661 genes were positively correlated with at least one other gene and 14,707 of 230,860 possible gene pairs were significantly positively correlated in both SPF and GF skin (Fig. 4a). Additionally, 605 of these 14,707 positively correlated gene pairs exhibited a significant change in correlation between the two colonization states, indicating an underlying change in modular connectivity profiles. Notably, *Loricrin*, which encodes a major component of the cornified envelope, and *Serpina12*, a serine protease inhibitor that has been implicated in the keratinocyte desquamation process [47, 48], are both significantly upregulated in the presence of commensal microbiota and are also significantly positively correlated in GF and SPF conditions (Fig. 4b). However, a significant decrease in the correlation coefficient between the two genes is observed in SPF compared to GF skin (Fig. 4b, q, < 0.05), suggesting an alteration in the gene networks controlling epidermal development in response to microbial colonization.

Genes with positive correlations in both colonization states (*n* = 661) were further scrutinized for shared transcription factor binding sites in oPOSSUM3 [49]. For improved resolution, these genes were also stratified by whether they were upregulated in GF (*n* = 196) or SPF (*n* = 465) skin. Twenty-eight total transcription factors (TF) were enriched in our positively correlated gene list (Fig. 4c, Additional file 10: Figure S5). Strikingly, Klf4, an important regulator of epidermal differentiation and barrier formation [50, 51], was the most significant TF across all three gene groupings, validating the relevance of our selected gene set.

Other significantly enriched TFs, such as SP-1 and AP-1, were more discriminatory of colonization status than Klf4. In our analysis, SP-1 was more significant in predicted regulation of GF compared to SPF genes, while AP-1 was predicted to be more significant in regulating SPF genes when considering the Fisher score metric (Fig. 4c). SP-1 has been implicated in regulating epidermal barrier function and, in conjunction with AP-1, regulates keratinocyte-specific gene expression in vitro [52]. Klf4 and SP-1 have predicted binding sites in both *Loricrin* and *Serpina12*, while AP-1 is predicted to only target *Loricrin*. Together, these findings suggest that the commensal microbiota differentially regulates underlying gene networks under the control of these TFs in the skin.

### DEGs under microbial regulation are common to the skin and gastrointestinal tract

**Fig. 5:**
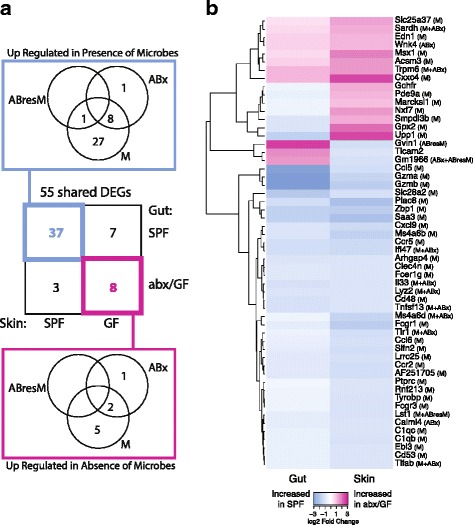
Comparison to published gut transcriptome dataset identifies shared DEGs under microbial regulation. **a** Venn diagrams highlighting 55 DEGs shared between skin and gut that are regulated by the microbiota. Gut transcriptome data was downloaded from a previously published study [23]. The center square identifies the total number of shared DEGs between the skin (*x*-axis) and gut (*y*-axis) datasets in each colonization category. The Venn diagrams highlight DEGs upregulated in the presence (blue, top) and absence (magenta, bottom) of microbiota, respectively, and whether these genes were differentially regulated in the gut in response to microbial colonization (M), colonization of antibiotic resistant microbes (ABresM), or direct effects of antibiotics on host tissue (ABx) or any combination of the above. **b** Heatmap showing log2 fold change of the 55 DEGs shared between the gut and skin datasets, with parenthesis next to gene names indicating whether these genes were differentially regulated in the gut in response to microbial colonization (M), colonization of antibiotic resistant microbes (ABresM), direct effects of antibiotics on host tissue (ABx), or any combination of the above

Modulation of gene expression by the gut microbiota has been extensively studied in gastrointestinal tissues [19–24]. To determine if genes and pathways are similarly regulated by microbial colonization in both the skin and gastrointestinal tract, we compared our dataset to a 2015 study that examined gene expression profiles of control (conventionally raised SPF) mice to GF mice and mice treated with antibiotics to deplete microbiota [23]. Our data shared 995 of these DEGs; 55 of which were significant with at least a twofold change in expression in both datasets (Fig. 5a, genes listed in Fig. 5b). For each gene, Morgun et al. attributed the observed differential expression in the gut tissue to direct effects of antibiotics on host tissue (ABx), depletion of normal microbiota in the gut (M), and/or growth of antibiotic resistant bacteria (ABresM). Compellingly, genes under microbial regulation in both the skin and GI tract were mainly attributed to the depletion of the normal microbiota rather than to side effects of antibiotic usage (Fig. 5a).

The 37 DEGs shared in the gut and skin that are upregulated during microbial colonization include genes related to the immune response, such as the complement cascade (*C1qc*, *C1qb*), cytokines and chemokines (*Il-33*, *Ccr2*, *Ccr5*, *Ccl5*, *Ccl6, Cxcl8*, *Cxcl9*), and toll-like receptors (*Tlr1*) (Fig. 5b). In particular, IL-33 has been implicated in multiple inflammatory disorders of both the skin and gut. Transgenic expression of IL-33 in murine skin causes spontaneous dermatitis to develop [53] and, in humans, may modulate filaggrin expression and thus skin barrier function [54]. IL-33 is also implicated in colitis in humans and in mouse models [55, 56], suggesting a general role for IL-33 signaling in the mediation of host-microbe interactions at epithelial barrier surfaces. Ebi3 is a subunit of the heterodimeric cytokine IL-27, which plays an essential role in regulating cellular proliferation during skin wound repair [57] but similarly mediates intestinal epithelial cell proliferation [58].

Eight genes were downregulated by the microbiota in both the skin and gut. Interestingly, three of these genes encode mitochondrial proteins involved in solute transport (*Slc25a37*), oxidative demethylation (*Sardh*), and acyl-CoA metabolism (*Acsm3*), suggesting conserved roles for the microbiota at both tissue types in cellular metabolism. Edn3, or endothelin 3, is the ligand for endothelial receptor type B (Ednrb), which is expressed by neural crest-derived lineages during development, namely melanocytes (in the skin) and enteric neurons (in the gut) [59]. Trpm6 and Wnk4 are both involved in ion transport in the colon [60], but their role in skin is unclear.

Ten genes were differentially expressed in opposite directions in the gut and skin in response to microbial colonization. Genes upregulated by microbiota in the skin but downregulated by microbiota in the gut, include very large interferon-inducible-GTPase (*Gvin1*), toll-like receptor adaptor molecule 2 *(Ticam2)*, and a predicted gene (*Gm1966*). Conversely, genes upregulated by microbiota in the gut but downregulated by microbiota in the skin, include GTP cyclohydrolase I feedback regulator (*Gchfr*), phosphodiesterase 9A (*Pde9a*), MARCKS-like 1 (*Marcksl1*), nuclear RNA export factor 7 (*Nxf7*), sphingomyelin phosphodiesterase, acid-like 3B (*Smpdl3b*), glutathione peroxidase 2 (*Gpx2*), and uridine phosphorylase 1 (*Upp1*). The discordant expression of these genes suggests distinct roles and/or requirements for tissue homeostasis and tolerance in the gut and skin.

## Discussion

As a barrier to the external environment, the skin must effectively orchestrate gene expression programs to establish host-microbe commensalism and maintain cutaneous barrier function. Here, by integrating microbiome research with transcriptional genomics, we investigated cutaneous gene expression profiles from GF and SPF mice to determine how the skin interprets exposure to the commensal microbiota on a genome-wide scale. We identified a previously supported role for the microbiota in regulating immune response pathways in the skin and more surprisingly revealed that the microbiota influences epidermal development and differentiation pathways. We also identified commonalities in the genes and pathways regulated by the microbiota in the gut and skin. Together, these findings provide novel insight for understanding the fundamental and diverse cutaneous functions imparted by the commensal microbiota and establish a critical resource for further exploration.

Previous work has established cell-type-, micro-organism- and pathway-specific roles for the skin microbiota in cutaneous immunity. For example, different skin resident microbes can control expression of antimicrobial peptides [61]. Cutaneous IL-1 signaling has also been shown to be augmented by the commensal microbiota, subsequently promoting effector T cell functions [6]. Commensal microbes are also responsible for accumulation of regulatory T cells via a Ccl20-Ccr6 axis in neonatal skin [15]. The work herein collectively confirms these findings at the transcriptome level, while revealing additional immune pathways and responses elicited by the skin microbiota.

Our high-throughput approaches revealed significant transcriptional differences in response to microbial colonization. Network analyses identified co-expressed gene modules in the cutaneous transcriptome. Particularly, two gene expression modules including a significant proportion of genes under microbial regulation contribute to the host-immune response. Upregulation of innate immunity genes in the presence of microbes could be associated with the higher levels of IL-1α observed in SPF compared to GF skin. It is important to note that these expression differences are not accompanied by an increase in overall inflammation, supporting the role of the microbiome in priming the cutaneous immune response.

Our data suggest an increase in the proliferative capacity of GF skin and the microbial regulation of genes in the EDC. The enrichment of DEGs in this syntenic and relatively gene-dense region may suggest some interaction between the microbiome, the epigenome, and other regulatory mechanisms. While previous work has shown that genes in the EDC are coordinately regulated [35], it is intriguing to hypothesize that this regulation may in part be modulated by the microbiota. We identified putative transcription factors associated with differentially expressed, differentially correlated genes. Although further investigation is required to elucidate the exact mechanisms, our data suggest that genes regulated by transcription factors such as Klf4, AP-1, and SP-1 may be regulated in a colonization-dependent manner. A similar phenomenon described in the gastrointestinal tract demonstrates that hundreds of genes under negative regulation by the transcription factor Hnf4 in zebrafish are microbially regulated, many of which were homologs of genes associated with human inflammatory bowel diseases [62].

Another noteworthy finding is the characterization of genes that are transcriptionally modulated by the microbiome in both the gut and the skin, suggesting that while microbiota across different tissues induce niche-specific gene expression changes, they also stimulate similar host-immune responses. A limitation of this study is that in this model system, it is not possible to separate the effects of gut microbiota from skin microbiota. It is possible that the gut microbiota influences processes at distal epithelia such as the skin, through intestinal absorption of microbiota-derived metabolites into the bloodstream or through effects on immune cell stimulation and/or programming [63]. Similarly, the skin microbiota may have physiological implications at distal sites, which were not investigated here. Additionally, although we used sex- and age-matched mice in this study and confirmed synchronized hair cycles by histology, endogenous host factors, such as sex, age, and hair cycle, should be further investigated for their potential to modify cutaneous host-microbe interactions. Finally, our study does not differentiate the effects of different species/strains of microbiota on transcriptional responses in the skin.

Our foundational approach focused on transcriptional responses to the whole microbial community colonizing conventionally raised SPF mice in comparison to GF mice. Our work provides a framework for further investigation into how specific microbial lineages, host-genetic variation, disease states, and environmental challenges influence microbially mediated gene expression in the skin.

## Conclusions

Our results suggest that the skin microbiome mediates two fundamental processes at the transcriptional level in the skin: the immune response and epidermal development and differentiation. The epidermal differentiation complex was enriched in differentially expressed genes, and differential gene correlation analysis suggests that genetic networks underlying epidermal barrier formation and differentiation are altered by the microbiota and regulated by transcription factors such as Klf4, AP-1, and SP-1.

## Methods

### Gene expression analysis

#### Library preparation

All mouse experiments were conducted under protocols approved by the University of Pennsylvania Institutional Animal Care and Use Committee. Mice were born as GF or SPF at the University of Pennsylvania and housed as such, 3-5 mice per cage, until they were euthanized for tissue harvest. The skin was collected from the dorsum of 8–10-week-old male C57BL/6 mice and stored in RNAlater. Mice were confirmed to have similar (telogen) hair cycle by histological analysis. Poly-A enriched RNA was isolated from harvested GF (*n* = 9) and SPF (*n* = 7) skin, and RNA-seq libraries were constructed using the unstranded TruSeq RNA Sample Prep Kit (Illumina). Consistent with ENCODE recommendations, libraries were sequenced on the Illumina HiSeq 2000 to obtain 100 bp paired-end reads per skin sample.

#### Alignment, filtering, and counting

Transcripts were aligned to the mouse reference genome GRCm38.p4 v9 [64], using STAR [27] in conjunction with AlignerBoost [28], and specifying a seed length of 25, 4% seed mismatch, 0% seed indels, 8% all mismatch, and 3% all indels. Reads mapping to numbered and sex chromosomes were retained. Read counts were generated using featureCount in the subread package [65] and counts to ribosomal RNA were removed. Reads were filtered in NOISeq [29, 30] using method 1, which removes features with a sum of expression values less than 1 count per million multiplied by the number of samples in the condition. Post filtering, TMM normalization was applied and technical batch effects associated with sequencing run were removed using ARSyNseq [31].

#### Weighted gene correlation network analysis

Filtered, normalized, and batch effect-corrected gene counts were input into the WGCNA R package [32]. A signed-hybrid network was constructed specifying the following parameters (power = 17, pamRespectsDendro = FALSE, minModuleSize = 30, reassignThreshold = 0, mergeCutHeight = 0.25). Gene ontology analysis of modules was performed by converting Ensembl gene IDs to Entrez gene IDs with biomaRt [66] and using the function “GOenrichmentAnalysis” (parameters: organism = “mouse,” nBestP = 5, ontologies = c(“BP”)).

#### Differential gene expression and gene ontology analysis

Differential gene expression was determined using NOISeqBIO (*q* = 0.9, equivalent to FDR adjusted *p* value of 0.1). Gene ontology (GO) [67] and KEGG pathway [68] analysis were performed using the R package GOSeq [69], and visualization was generated with REVIGO [70], allowing medium similarity, using the “*Mus musculus*” database, and the SimRel similarity measure.

#### Differential gene correlation and TFBS analysis

Gene correlation analysis was performed on all 2820 DEGs with the DGCA R package, using default parameters unless otherwise specified [46]. Initially, genes were filtered for low central tendency, retaining only genes with average expression levels in the 75th percentile or above in all tested genes. Genes were further filtered for dispersion, retaining only genes with moderate to high dispersion of expression values (above the 30th percentile). The differential correlation analysis was performed on all possible pairs using Pearson’s correlation coefficient. Significance was determined through empirical *p* values derived from *Z* scores obtained in comparing the correlation values of the original expression data with correlation values of permuted expression data; 10 permutations were performed. Only genes with positive correlations in both colonization states were further considered. Prediction of overrepresented transcription factor binding sites in the positive/positive correlated genes was performed using a single site analysis with default parameters in oPOSSUM3 [49]. Significance was assessed using Fisher scores (significant when > 1 standard deviation above the mean) and *Z* scores (significant when > 2 standard deviations above the mean). Differences in the relative significance levels between the two scoring methods result from the different parameters and sample distributions used to calculate each score. Additionally, *Z* scores consider the total number of TFBS in a gene set, while Fisher scores only consider the number of genes in a set containing at least one TFBS.

#### Comparisons to previously published datasets

Significantly differentially expressed genes with at least a twofold change in expression from a gut microbiome dataset [23] were downloaded from published supplementary data and imported into R.

### Cellular characterization

#### Histology and immunofluorescence

Skin biopsies were collected from the dorsal side of SPF and GF mice, fixed in 10% (*w*/*v*) formalin, embedded in paraffin, and sectioned at 6 μm. Tissue sections were stained with hematoxylin and eosin to characterize epidermal thickness or with toluidine blue to identify mast cells. For immunofluorescence, sections were deparaffinized with xylene and rehydrated in downgraded alcohol. Heat-inactivated antigen retrieval was performed by incubating the tissue sections in 10-mM sodium citrate buffer, pH 6.0, and subsequently washing the sections with a PBS/0.2% Triton solution. Tissue sections were blocked with 10% (*v*/*v*) normal goat serum for 2 h at room temperature. After blocking, sections were incubated with a primary antibody. The antibodies that were used include anti-mouse Keratin 6A (Biolegend), anti-mouse Loricrin (Biolegend), anti-mouse CD3 (Abcam), and anti-mouse Ki67 (Abcam). Following multiple washes, secondary antibodies, goat anti-rabbit IgG-Alexa, and goat anti-mouse-Alexa 555 were applied for 1 h at room temperature and then washed. Slides were mounted with prolong DAPI (Molecular Probes) and examined under a fluorescent microscope (Leica DM550B). Positive-stained cells were counted in five fields per tissue section at × 400 magnification, three tissue sections per mouse, and three mice per group.

#### Tissue processing and flow cytometry

Skin biopsies were collected from the dorsal side of 5 SPF and 5 GF mice. A section of skin was harvested from the dorsum of the mice following hair removal with an electric trimmer equipped with a two-hole precision blade (Wahl) and treatment with a hair removal lotion (Nair). Skin sections were then minced with a sterile scalpel blade into ~ 2-mm sections and incubated in 5 mL of RPMI containing 12.5 mg/mL of Liberase TL (Roche) and 100 μg/mL of DNAse I (Sigma-Aldrich) for 120 min with vortexing every 30 min. The resulting single cell solution was passed through a 40-μm cell strainer and resuspended in cRPMI. For analysis of surface markers and intracellular cytokines, cells were incubated for 4 h with 10 μg/mL of brefeldin A, 50 ng/mL of PMA, and 500 ng/mL ionomycin (Sigma-Aldrich). Before staining, cells were incubated with anti-mouse CD16/CD32 mouse Fc block (eBioscience) and 10% rat-IgG in PBS containing 0.1% BSA. Cells were stained for dead cells with LIVE/DEAD Fixable Aqua Dead Cell Stain Kit (Molecular Probes) and surface markers (CD4 [eBioscience, clone RM4-5], CD8β [BioLegend, clone YTS156.7.7], CD45 [eBioscience, clone 30-F11], TCRγδ [BD Biosciences, clone GL3], Ly6G [eBioscience, clone 1A8-Ly6g], Ly6C [BD Biosciences, clone AL-21], CD11b [eBioscience, clone M1/70], CD11c [eBiosciences, clone N418], F4/80 [eBioscience, clone BM8]) followed by fixation with 2% of formaldehyde and permeablization with 0.2% saponin/PBS. Intracellular cytokine staining was performed for pro-IL-1β (eBioscience, clone NJTEN3), IL-17 (eBioscience, clone eBio17B7). The data were collected using LSRII flow cytometer (BD) and analyzed using FlowJo software (Tree Star).

## Additional files

Additional file 1: Table S1. Sample summary statistics. Rows contain the 16 samples analyzed; with columns containing associated sequencing statistics and metadata. (XLSX 56 kb)

Additional file 2: Figure S1. Quality control of RNA-sequencing data. (A) Mean quality score per base for each of the 16 samples. (B) Number of reads mapping to the mouse reference genome for each sample. (C) Relative abundance of reads mapping to each biotype. (D) Percentage of the genome covered by mapped reads per sample. (EPS 1354 kb)

Additional file 3: Dataset S1. Results from differential expression analysis. Rows contain the 15,448 features analyzed. Columns contain Ensembl feature id, mean expression of GF samples, mean expression of SPF samples, the NOISeq differential expression statistic theta, the probability of differential expression (equal to 1-FDR-corrected *p* value when using NOISeqBio, DEGs defined as those with prob. > 0.9), the log_2_ fold change in expression (upregulated in GF > 0, downregulated in GF < 0), feature length, chromosome, feature start and end coordinates, feature biotype, and feature symbol. (XLSX 2289 kb)

Additional file 4: Figure S2. Batch effect correction improves dataset quality. NMDS plot (A) based on filtered, normalized read counts, and (B) on filtered, normalized, batch effect-corrected read counts from each sample, showing that batch effect correction improves dataset quality. Each point represents a single sample, with the color indicating condition (blue = SPF, magenta = GF) and shape indicating sequencing run. ARSyNseq was run with the following parameters (factor = “RunName”, batch = TRUE, norm = “n”, logtransf = FALSE). (EPS 642 kb)

Additional file 5: Figure S3. WGCNA identifies cutaneous gene modules. (A) Hierarchical clustering of samples prior to network generation (B) thresholding analysis, showing scale-free properties of the network with a chosen soft threshold power of 17. Yellow line indicates an *R*^2^ value of 0.8, orange of 0.85, and red of 0.9. (C) WGCNA cluster dendrogram of genes in our dataset, with the module membership highlighted below the dendrogram. Gray indicates genes not belonging to any of the determined modules. Dendrogram represents hierarchical clustering of eigengene modules in relation to each other and colonization condition. (D) Correlation of each module to metadata. For each comparison, the rho value is provided above the *p* value in parentheses. No modules are significantly correlated with sequencing run. The color of each box in the “Colonization Condition” column indicates the strength of the positive correlation with SPF (blue) or GF (magenta) states. (EPS 6217 kb)

Additional file 6 Dataset S2. Results from WGCNA analysis. Rows contain the features contained in each WGCNA module. Columns contain Ensembl feature id, gene symbol, module name, module color membership, gene significance (GS) for colonization condition (defined as the absolute value of the correlation between the feature and metadata) and associated *p* value, and module membership (MM) for each module (defined as the correlation of the module eigengene and the gene expression profile) and associated *p* value, the Entrez gene ID used for gene ontology analysis, and the differential gene expression status. (XLSX 8459 kb)

Additional file 7: Table S2. WGCNA gene module characterization. Top 5 significantly enriched biological process gene ontology terms (Bonferroni *p* < 0.05) associated with each WGCNA module. (XLSX 47 kb)

Additional file 8 Dataset S3. Results from gene ontology analysis. Each worksheet contains differential expression analysis results for features in the mentioned gene ontology categories. Blue and orange cells indicate significant DEGs with and without a twofold change difference in expression, respectively. (XLSX 367 kb)

Additional file 9: Figure S4. Analysis of skin immune cell populations supports gene expression findings. (A) Toluidine blue staining for mast cells. (B) Immunofluorescence staining of CD3, a pan T cell marker. Significance testing was performed on an aggregate of three experiments with *n* = 3 GF and SPF mice each. (C) Flow cytometry analysis of GF and SPF (*n* = 5 each) of IL-1α and IL-1β production by cell subset. Comparisons that are significantly different with a *p* value < 0.05 are denoted with * and those with a *p* value < 0.01 with **. (D) Barplots showing normalized gene expression values for *IL-1α* and *IL-1β*. Lines depict standard error and padj represents the FDR-corrected *p* value (1-prob) calculated by NOISeqBio. (E) Boxplot of normalized gene expression of terminal differentiation markers *Krt1* and *Krt14*, with padj indicating the FDR-corrected *p* value (1-prob) calculated by NOISeqBio. (EPS 85855 kb)

Additional file 10: Figure S5. DGCA analysis identified significantly correlated DEGs that share potential transcription factor binding sites. Analysis with oppossum3 identified enriched transcription factors in positively correlated DGCA gene sets, using *Z* scores to assess significance. The *y*-axis identifies significant transcription factors, while *x*-axis represents the significance metric, with higher values indicating greater significance, and the shape indicating whether the metric score was 1 or 2 standard deviations (SD) above the mean. *Z* scores are significant when greater than 2 SD above the mean. Size of each point reflects the percentage of all DGCA +/+ DEGs containing a binding region for each TF and color indicates colonization status of the DGCA +/+ DEGs. (EPS 1582 kb)

## Abbreviations

DEG: Differentially expressed gene
DGCA: Differential Gene Correlation Analysis
EDC: Epidermal differentiation complex
FDR: False discovery rate
GF: Germ free
GO: Gene ontology
IL-1: Interleukin 1
KEGG: Kyoto Encyclopedia of Genes and Genomes
NMDS: Non-metric multidimensional scaling
SPF: Specific pathogen free
TF: Transcription factor
WGCNA: Weighted gene network analysis
